# Supplementary material for: Anti SARS-CoV-2 Monoclonal Antibodies in Pre-Exposure or Post-Exposure in No- or Weak Responder to Vaccine Kidney Transplant Recipients: Is One Strategy Better than Another?
Source: Viruses. 2024 Feb 29;16(3):381. doi: 10.3390/v16030381 (PMC10975193; doi:10.3390/v16030381)
Supplement: Supplementary file 1 [file viruses-16-00381-s001.zip › viruses-2832731-supplementary.pdf]

Table S1. Baseline patients characteristics.

Age-Years	56,7 ± 14,1
Male sex, n (%)	104 (58)
Time from kidney transplantation, median (range)	64,6 (33,5-146)
Kidney transplantation <3 months, n (%)	2 (1,1)
Transplant number	1,2
**Induction therapy for KT n (%):**	
ATG	83 (46,4)
Anti-R-IL2	92 (51,4)
Unkown	4 (2,2)
eGFR- mL/min/1.73m^2	44 ± 20,2
Body mass index, kg/m^2	27,0 ± 5,0
**Nephropathy :**	
Other cause, n (%)	1 (0,6)
Diabetes, n (%)	8 (4,5)
Glomerulonephritis, n (%)	74 (41,3)
Unkown cause, n (%)	14 (7,8)
Interstitial nephropathy, n (%)	14 (7,8)
Hypertension, n (%)	12 (6,7)
Malformative uropathy, n (%)	20 (11,1)
Polycystic kidney, n (%)	36 (20)
**Comorbidities :**	
Hypertension, n (%)	146 (81,6)
RAS blockers, n (%)	87 (59,6)
Diabetes, n (%)	46 (25,6)
Insulin, n (%)	28 (60,8)
Cardiac disease, n (%)	63 (35,2)
Vascular disease, n (%)	16 (8,9)
Respiratory disease, n (%)	26 (14,5)
Cancer, n (%)	35 (19,5)
Active smoking, n (%)	18 (10)
Former smoker, n (%)	61 (34)
Cirrhosis, n (%)	9 (5)
**Immunosupressive drugs at inclusion :**	
Mycophenolic acid, n (%)	154 (86)
Azathioprine, n (%)	12 (6,7)
Ciclosporin, n (%)	16 (8,9)
mTOR inhibitor, n (%)	3 (1,7)
Tacrolimus, n (%)	128 (71,5)
Belatacept, n (%)	29 (16,2)
Corticosteroids, n (%)	101 (56,4)

Data are given as mean ± SD, median ± IQR, n nomber ; KT, kidney transplant ; mTOR, mammalian target of rapamycin ; ATG, antithyroglobulin ; RAS, renin-angiotensin system.

Table S2. COVID-19 disease presentation, management and clinical outcomes.

**Symptoms, n (%)** : n = 170	144 (80,4)
Cough, n (%)	75 (44,1)
Fever, n (%)	50 (29,4)
Rhinitis, n (%)	49 (28,8)
Asthenia, n (%)	46 (27)
Myalgia, n (%)	27 (15,9)
Diarrhea, n (%)	27 (15,9)
Dyspnea, n (%)	25 (14,7)
Odynophagia, n (%)	20 (11,7)
Headaches, n (%)	19 (11,2)
**Imagery, n (%) :**	28 (15,6)
Radiography, n (%)	7 (25)
Pulmonary computed tomography, n (%)	21 (75)
**Antibiotic, n (%)** :	21 (11,7)
Piperacillin/tazobactam, n (%)	9 (42,8)
Rovamycin, n (%)	7 (33,3)
Ceftriaxone, n (%)	6 (28,6)
Daptomycin, n (%)	1 (4,7)
Cefepime, n (%)	1 (4,7)
Quinolone, n (%)	4 (19)
Amoxicilline clavulanate, n (%)	3 (14)
Amoxicillin, n (%)	1 (4,7)
Caspofungin, n (%)	1 (4,7)
Metronidazole, n (%)	1 (4,7)
Dexamethasone, n (%)	14 (7,8)
Convalescent plasma, n (%)	5 (2,8)
Oxygen therapy, n (%)	21 (11,7)
Acute renal injury, n (%)	23 (12,9)
Hospitalization, n (%)	32 (17,9)
Hospitalization in intensive care unit, n (%)	7 (3,9)
Death, n (%)	6 (3,3)

n, number.
